# Risk factors for malunion of distal tibia fractures treated by intramedullary nailing

**DOI:** 10.1186/s13018-023-04472-3

**Published:** 2024-01-03

**Authors:** Selim Daas, Mohamed Jlidi, Nahla Baghdadi, Walid Bouaicha, Karim Mallek, Mouldi Lamouchi, Adel Khorbi

**Affiliations:** 1 Orthopedics and Traumatology Department, Mohamed Taher Maamouri Hospital, AFH City, Mrezgua, 8050 Nabeul, Nabeul Tunisia; 2 Faculty of Medicine of Tunis, University of Tunis El Manar, Tunis, Tunisia

**Keywords:** Intramedullary nailing, Malunion, Risk factors, Surgical complications

## Abstract

**Background:**

The treatment of distal tibia fractures (DTF) has historically been a difficult challenge for orthopedic surgeons because of the particular characteristics of this anatomical region. Intra medullary nailing (IMN) remains the best treatment option. However, achieving and maintaining perfect reduction and stable fixation with IMN can be technically challenging due to the large medullary cavity within a short distal fragment.

The aim of our study is to determine the risk factors for malunion in DTF treated with IMN.

**Methods:**

It is a retrospective study including DTF treated surgically by IMN in the Orthopedics and Trauma Department at a tertiary hospital over a period of 7 years. The quality of reduction was evaluated by radiological assessment of the antero-posterior (AP) and lateral views of the tibia and ankle at the last follow-up.

**Results:**

Our series included 90 patients with an average age of 44.8 years. Sex-ratio was 2.6. Tobacco use was reported in 35.6% of the patients. Diabetes was present in 11.1% of the patients, and 12.2% of them had open fractures. According to the OTA/AO classification, the majority of injuries were classified as type A1 (76.7%). Fibula fractures were present in 86.7% of cases.

The mean follow-up was 48 months. Malunion occurred in 13 cases.

Based on the univariate analysis, smoking and dynamic fixation were significantly associated with malunion. In the multiple logistic regression analysis, dynamic fixation was found to be a significant factor that increased the risk of malunion by 7.5 times.

**Conclusion:**

Neither patient demographics nor fracture characteristics were risk factors for malunion. Nevertheless, it should be noted that dynamic nailing must be avoided as it is associated with a higher risk of malunion. Furthermore, one to two medial to lateral distal locking screws provide sufficient stability without the need for additional fibular fixation.

***Trial registration*:**

Not applicable.

## Introduction

Distal tibia fractures (DTF) are common injuries. Their incidence rate is reported to be increasing due to the expansion of traffic road accidents [1]. Muller defined these fractures as being contained within a square whose sides are equal to the widest part of the epiphysis [2]. Fractures occurring in this region are difficult to treat. In fact, due to the proximity to the ankle joint, the limited muscle cover and the poor blood supply. These fractures are at a high risk of complications such as malunion, nonunion, infection and post traumatic osteoarthritis [3, 4].

Several treatment modalities are currently used, including conservative management, open reduction and internal fixation (ORIF) using plates, minimally invasive plate osteosynthesis (MIPO) and external fixation [5]. Non-operative management indications are currently limited to strictly undisplaced fracture and in patients with contra indications to surgery [6]. ORIF with plate and screws is associated with high infection rates leading to the development of minimally invasive techniques. External fixation techniques are linked with pin track sepsis, malunion and algoneurodystrophy [5].

Intra medullary nailing (IMN) remains the gold standard of treatment [7].This surgical procedure provides high union rates with fewer cutaneous complications and surgical wound infections [8]. However, achieving and maintaining perfect reduction and stable fixation with IMN can be technically challenging due to the large medullary cavity within a short distal fragment. This explains the high rates of malunion often reported after this treatment modality, sometimes reaching 50% of the cases in some series [9, 10].

Several risk factors for malunion in DTF are reported in the literature, most of them related to the role of fibular fixation in maintaining alignment [11–13]. Only few studies have included other factors related to the patient, fracture characteristics and surgical management [8, 11].

The aim of our study is to determine the risk factors for malunion in DTF treated with IMN.

## Material and methods

This is a retrospective study including DTF treated surgically by IMN in the Orthopedics and Trauma Department at a tertiary hospital in the north-west region of Tunisia, over a period of 7 years extending from January 2015 to December 2021.

**Fig. 1:**
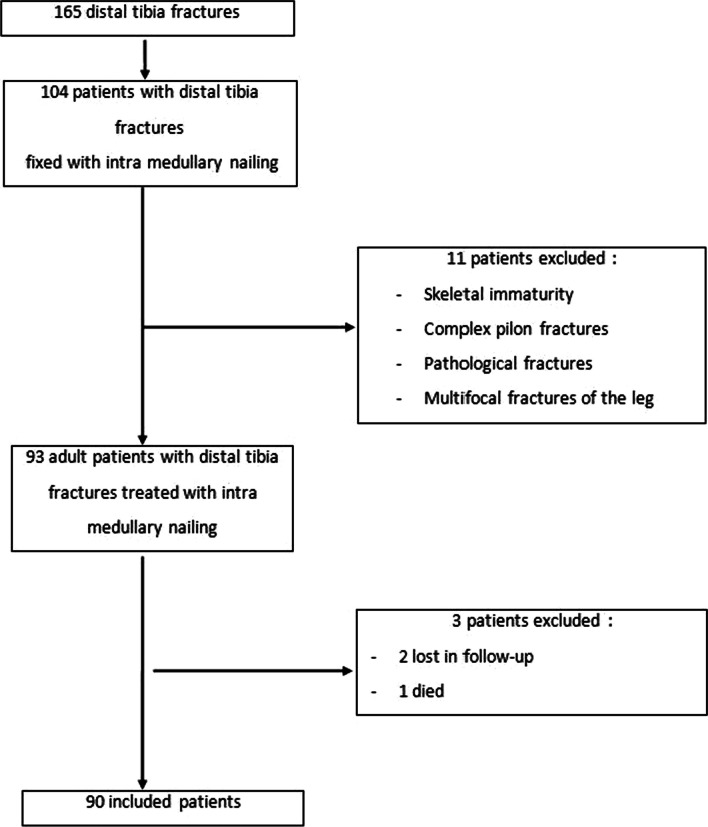
Flowchart of inclusion of patients with distal tibial fractures in the study

The files of all adult patients treated surgically for DTF using an IMN during this period were included. Ninety files met our inclusion and exclusion criteria and were enrolled for the study (Fig. 1). We used the Association of Osteosynthesis/Orthopedic Trauma Association (AO/OTA) classification system [14] of tibial pilon fractures. A minimum follow-up of 12 months was recommended.

Exclusion criteria were: patients younger than 18 years, pathological fractures, pilon fractures, other fixation procedures, other complications (infection, non-union), incomplete records and follow-up time of less than 12 months.

Open fractures were classified according to the Gustilo-Anderson classification [15].

The patients’ demographics were recorded: age, gender, tobacco use, history of diabetes, mechanism and etiology of injury, side of injury, fracture pattern and any associated neurological or vascular injury.

All the patients were reviewed at outpatient department at three weeks post-operatively, six weeks, three months, six months and yearly thereafter.

## Radiological evaluation

We assessed the quality of reduction on antero-posterior (AP) and lateral views of the tibia and ankle at the last follow-up.

Malunion was diagnosed when [16]:

- Coronal (varus or valgus) deformity > 5°
- Sagittal deformity (anterior or posterior angulation) > 10°
- Shortening > 10 mm
- Rotational deformity > 10°

## Statistical analysis

The statistical analysis was performed by an independent statistician using SPSS version 25.0. Firstly, a univariate analysis was conducted to assess the association between potential risk factors and malunion. Continuous variables were analyzed using independent samples t tests, while categorical variables were analyzed using and Chi-square and Fischer’s exact tests. At a second stage, all variables with a univariate *p* value of ≤ 0.10 were considered eligible for inclusion in a multiple logistic regression analysis. In the final model, significant factors were defined as those variables that displayed a *p*-value of *p* ≤ 0.05. The odds ratio (OR) and 95% confidence intervals (CI) were then reported to determine the strength and direction of the association between the significant factors and malunion.

## Results

### Descriptive results

The average age of the patients was 44.8 years (range from 19 to 91 years). Men were more affected than women with a sex-ratio of 2.6 (65 males and 25 females). Tobacco use was reported in 35.6% of the patients. Diabetes was present in 11.1% of the patients. The most common etiology was road traffic accidents accounting for half of the cases (55.6%), followed by domestic falls (33.4% of cases). Seven patients had associated injuries. The right side was most commonly affected (62.2%). Eleven patients in our study had open fractures (12.2% of the cases). Among these, 10 were type I according to Gustilo classification (11.1%) and one type II (1.1%). No neurological or vascular injuries were reported.

According to the OTA/AO classification, the majority of injuries were classified as type A1 (76.7%) followed by type A2 (15.6%). Type A3 fractures were noted in only 7.8% of the cases. A simple articular involvement was associated in 8 cases (8.9%). Fibula fractures were present in 86.7% of the cases. The most frequent location of the fibula fracture line was above the syndesmosis (50 cases), twenty of which were located at the neck of the fibula.

**Fig. 2:**
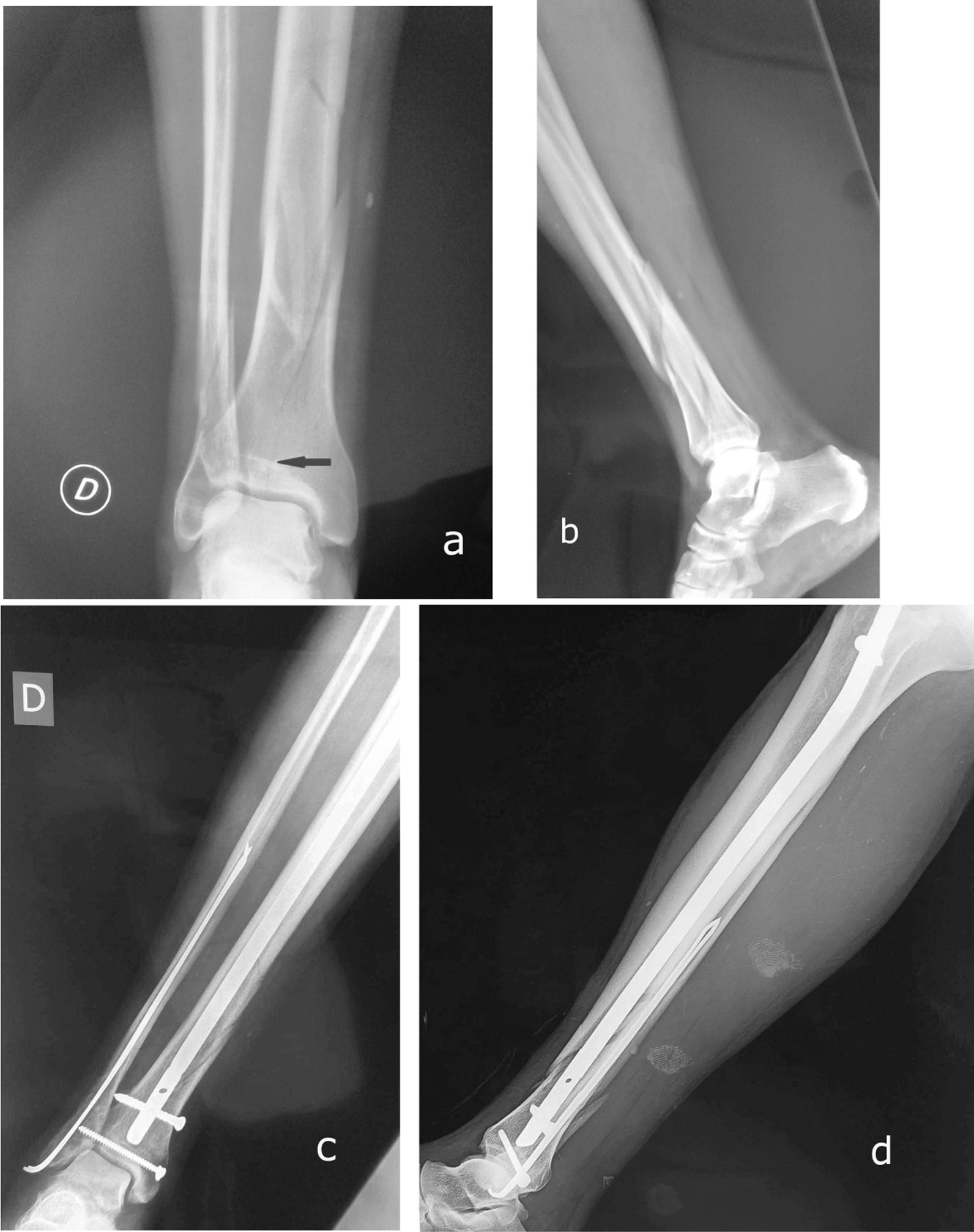
AP and lateral view of the left ankle (**a** and **b**) showing quart distal fracture type A3 according to AO-OTA classification with a simple articular involvement (arrow). This fracture treated with intramedullary nailing associated an additional screw fixation and fibula fixation by pin (**b** and **c**)

The mean waiting time to surgery was 28.04 h (range from 3 h to 6 days). The mean operating time was 81.06 min (range from 30 to 145 min). All patients were operated by IMN through an anterior infra patellar approach. The type of nail interlocking was static in 84.4% of the cases and dynamic in the rest. Tibial nails of 9 mm and 10 mm diameters were equally used (45 cases each). Distal locking screws were inserted in all cases with the use of one screw in 90% of the cases and two screws in 10%. The fibula fracture was fixed in 9% of the cases. Additional lag screw fixation was performed in 5 cases with a simple fracture line articular extension (Fig. 2).

## Radiological results

**Fig. 3:**
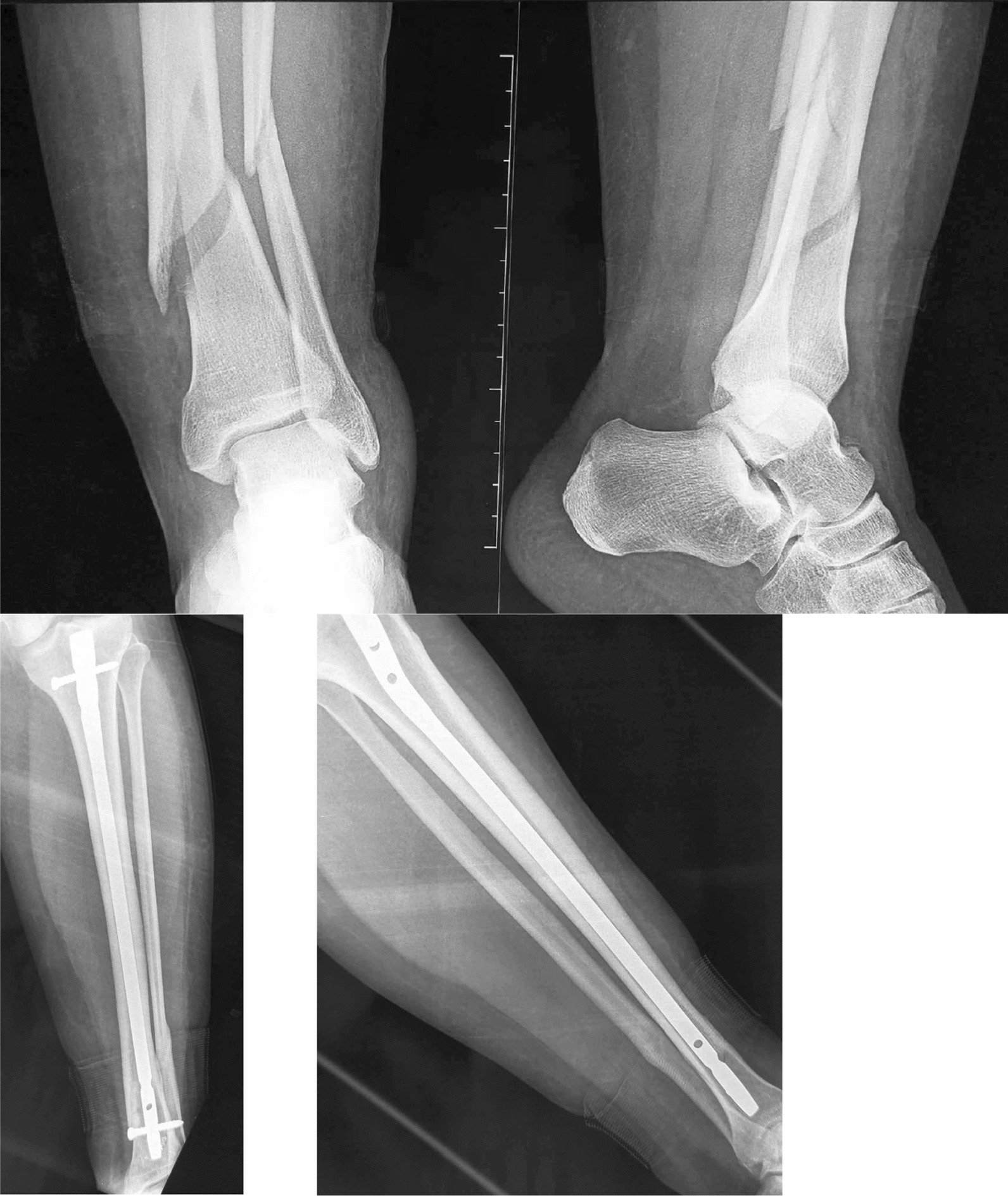
Distal tibia fracture, treated by intramedullary nailing, healed with good alignment

The mean follow-up was 48 months (range from 18 months to 8 years). All fractures in our study ultimately healed, with an average healing time of 4.3 months (range from 45 days to 12 months) (Fig. 3). Delayed union was observed in 11.1% of the patients.

**Fig. 4:**
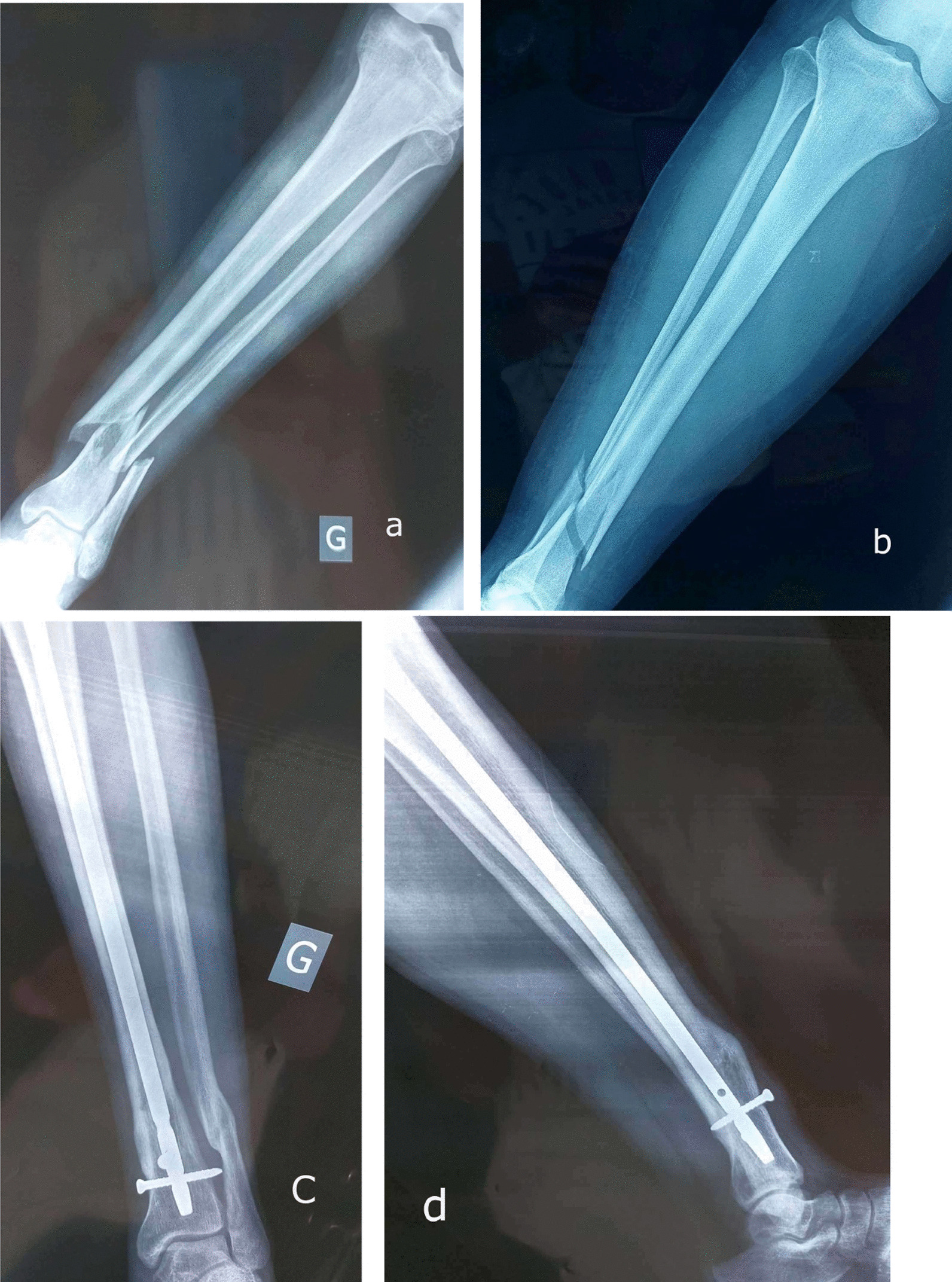
Distal tibia fracture associated a fibula fracture (**a** and **b**) treated by intramedullary nailing with a double block screws with an evolution toward malunion

At the last follow-up, malunion occurred in 13 cases (14.4%). Among these cases, there were seven cases of varus misalignment, one case of associated limb shortening, one case of valgus misalignment and four cases of apex anterior angulation including one associated external rotation (Fig. 4).

## Risk factors for malunion

Based on the univariate analysis, age, gender, diabetes history, mechanism of injury, open fracture, fracture severity, nail diameter, number of distal locking screws and fibula fixation were not found to be significantly associated with malunion (Tables 1, 2 and 3). However, smoking and dynamic fixation were associated with the occurrence of this complication.

**Table 1:** Patient Demographics factors for malunion

Variable	Malunion (*N* = 13)	No malunion (*N* = 77)	OR (95% CI)	*P* value
*Age*				
< 50 years	7	50	1.5 (0.48–5.2)	0.31
> 50 years	6	27		
Gender				
Male	12	53	5.4 (0.66–44.20)	0.07
Female	1	24		
*Diabetes*				
No	10	69	2.5 (0.58–11.40)	0.19
Yes	3	8		
Tobacco use				
No	5	52	3.3 (1–11.21)	0.04*
Yes	8	25		
Mechanism				
High energy	8	42	0.7 (0.22–2.5)	0.4
Low energy	5	35		

*OR: Odds ratio; CI: Confidence Interval*

*The threshold of statistical significance was set at 5%

**Table 2:** Fracture Characteristics factors for malunion

Variable	Malunion	No Malunion	OR (CI 95%)	*P* value
*Open fracture*				
No	12	67	0.5 (0.06–4.77)	0.5
Yes	1	10		
*Fracture type*				
A1/A2	12	71	1 (0.1–8.93)	0.7
A3	1	6		
*Fibula fracture*				
No	0	12	N/A	0.13
Yes	13	65		

*OR: Odds ratio; CI: Confidence Interval*

**Table 3:** Surgical characteristics factors for malunion

Variable	Malunion (*N* = 13)	No Malunion (*N* = 77)	OR (CI 95%)	*P* value
*Nail diameter*				
9mm	6	44	1.5 (0.47–5.06)	0.33
10mm	7	33		
*Nail locking type*				
Static	7	69	7.4 (2–27.49)	0.004*
Dynamic	6	8		
*Distal locking*				
1 screw	11	70	1.8 (0.33–9.9)	0.38
2 screws	2	7		
*Fibula fixation*				
No	13	70	N/A	0.26
Yes	0	7		

*OR: Odds ratio; CI: Confidence Interval*

*The threshold of statistical significance was set at 5%

In the multiple logistic regression analysis, there was no significant interaction identified between gender and tobacco use in relation to the occurrence of malunion. However, there was a non-significant trend toward a three times higher risk in male patients and a two and a half higher risk in smokers. On the other hand, dynamic fixation was found to be a significant factor that increased the risk of malunion by 7.5 times (Table 4).

**Table 4:** Multiple regression analysis—Tobacco use and type of fixation

Variable	OR (95% CI)	*P* value
*Gender*		
Female	Reference	0.3
Male	3.2 (0.31–33.93)	
*Tobacco use*		
No	Reference	0.2
Yes	2.5 (0.60–10.98)	
*Mounting type*		
Static	Reference	0.004*
Dynamic	7.5 (1.88–30.39)	

*OR: Odds ratio; CI: Confidence Interval*

^*^The threshold of statistical significance was set at 5%

## Discussion

Distal tibia fractures are serious injuries associated with a high risk of complications. In recent series, malunion rates following IMN varied widely and have been reported to be as high as 23% [9, 10, 17]. In our study, it was 14.4% which was consistent with previous literature on the subject.

Based on our multiple regression analysis, dynamic nailing was identified as the only independent risk factor for malunion, with a 7.5 times higher risk. Patients’ demographics, fracture characteristics, number of distal locking screws and the absence of fibular fixation were not found to be related factors.

In the study by Egol et al. [18], factors associated with insufficient reduction were examined immediately after surgery and at the last follow-up. He found that younger age was the only factor related to immediate misalignment after tibia IMN. However, at 12 weeks or more after surgery, the loss of reduction was not statistically associated with patient demographics (age, gender) or fracture characteristics (fracture type, fibula fracture). Inserting two medial to lateral distal locking screws appeared to be protective against malreduction. However, this factor did not reach statistical significance.

The number and orientation of locking screws in the treatment of DTF are still subject to debate. It is generally recommended to use a minimum of two screws to improve stability and control shortening and rotation, as well as to maintain alignment in the coronal and sagittal planes [3, 19]. Chan et al. [20] conducted a biomechanical study that investigated the effect of the number and positioning of distal interlocking screws after IMN of DTF. They found that three distal locking screws improved construct stiffness while reducing inter-fragmentary motion compared to using two screws only. According to this conclusion, one would expect higher malunion rates in the group of single distal locking screw compared to double distal locking screws. However, this was not the case in our series as the malunion rates were 13.6% and 28.5%, respectively; this difference was not statistically significant though.

Our results are corroborated by those of Fan et al. [21] who, in a series of 20 metaphyseal fractures treated by IMN with either one or two distal locking screws, found that both configurations secured inter-fragmental fixation and provided sufficient rigid stability resulting in an uneventful union. Furthermore, Kruppa et al. [22] compared three configurations (one, two or three distal locking screws) and found no significant difference in final alignment between the three groups.

Regarding the distal locking screws orientation, newer generation nail designs have been developed that offer more options for screw insertions. In these nails, locking holes are close to the tip of the nail and allow for multidirectional screw insertion offering surgeons various configuration options. As an example, the EXPERT® tibial nail provides four different distal locking options with two medial to lateral holes, one anterior to posterior and one oblique hole. Despite these advancements, many authors still prefer the two medial to lateral locking option [18, 23, 24].

A biomechanical study conducted by Lucas et al. [24]; it was found that adding a single anterior to posterior or oblique screw to a medial to lateral screw did not result in a superior stability compared to the use of two medial to lateral screws alone.

Since other multi-planar configurations did not demonstrate biomechanical superiority over the medial to lateral locking option, it seems reasonable for surgeons to use one or two medial to lateral screws as it is easier to insert with minimal risk for damaging the tibialis anterior artery and the extensor tendons to the foot [25].

Fibula fractures are commonly associated with DTF. When involving the syndesmosis, fibular fixation was highly recommended [8, 26]. The potential benefits of fibular osteosynthesis in these cases, especially in terms of stability, have not been clearly established.

Two biomechanical cadaveric studies have investigated the impact of additional fibular fixation. They found that fibular fixation only increased resistance to torsion forces, without adding significant coronal and sagittal planes stability [11, 27].

In clinical studies, the findings regarding the role of fibular fixation in distal tibia fractures are variable and sometimes contradictory. Some authors have found that failing to stabilize the fibular fracture was a risk factor for initial malreduction as well as secondary malunion [8, 12, 18]. A recent meta-analysis of four studies concluded that fibular fixation was significantly associated with a lower risk of mal-alignment [11]. The authors believed that their results only apply to classic IMN with two distal locking screws, as they suggested that modern tibial nails with more than two distal locking screws may provide sufficient stability regardless of concomitant fibular fixation.

In our series, only a few fibula fractures were fixed (7 out of 78). As a result, we could not find a statistically significant correlation between fibular fixation and the occurrence of malunion. However, it is worth noting that the rate of malunion was significantly higher when fibular fractures were not fixed (18.3% vs 0) with no significant difference.

Unlike our series, De Giacomo et al. [19] reported an overall low rate of malunion accounting for 3% in a large series of 122 DTF treated with IMN without fibular fixation. The authors attributed the success of their treatment approach to the rigorous insertion technique of the nail, emphasizing the importance of adequate fluoroscopic assessment of the quality of the reduction in both planes before inserting the nail and placing the distal locking screws. Based on their findings, they concluded that using modern intra medullary nailing with standard two medial to lateral distal locking screws can afford sufficient stability, rendering fibular fixation unnecessary.

Similarly, the randomized study conducted by Rouhani et al. [28] and the meta-analysis carried out by Li et al. [13] supported these findings. These authors did not find any advantages in associating a fibular fixation in DTF.

All these studies provide evidence that challenges the necessity of fibular fixation in certain cases, suggesting that modern IMN techniques and two medial to lateral distal locking screws may provide adequate stability without the need for additional fibular fixation.

Finally, inserting blocking screws is a useful technical artifact in DTF nailing procedure. These screws can help narrow the diameter of the medullary canal, reducing the mismatch with the diameter of the intra medullary nail and potentially correcting a varus or valgus deformity.

When DTF is associated with a severe osteoarthritis of the tibio-talar and subtalar joints, both problems can be addressed in a single procedure using a minimally invasive surgical technique providing a tibio-talo-calcaneal arthrodesis and fracture fixation using a retrograde IMN as described by Carlo Biz [29].

Our study reports on a large series of DTF treated by IMN focusing on one specific complication with a consistent statistical analysis. However, it remains a single center retrospective study with various surgeons doing the procedures.

## Conclusion

Malunion is a common complication following IMN of DTF. This is primarily attributed to the wide diameter of the medullary canal in the distal tibial metaphysis, which poses challenges in obtaining and maintaining proper reduction. Our data suggest that there is no significant correlation between patients’ demographics or fracture characteristics and the occurrence of malunion. Nevertheless, it should be noted that intramedullary nailing with dynamic interlocking must be avoided as it is associated with a high risk of malunion.

Our findings support the fact that one to two medial to lateral distal locking screws provide sufficient stability rendering an additional fibular fixation unnecessary.

## Data Availability

Datasets used can be accessed by correspond author.
